# Flexible Touch Sensors Made of Two Layers of Printed Conductive Flexible Adhesives

**DOI:** 10.3390/s16091515

**Published:** 2016-09-16

**Authors:** Sungwon Seo, Seonggi Kim, Jiyeon Jung, Rujun Ma, Seunghyun Baik, Hyungpil Moon

**Affiliations:** 1 School of Mechanical Engineering, Sungkyunkwan University, Suwon 440-746, Korea; ssw0536@skku.edu (S.S.); choucreme85@kimm.re.kr (S.K.); sbaik@me.skku.ac.kr (S.B.); 2 Department of Nature-Inspired Nano Convergence Systems, Korea Institute of Machinery and Materials, Daejeon 34103, Korea; 3 Hyundai Motor Company, Hwaseong 440-130, Korea; lamjiyeon@hyundai.com; 4 Department of Energy Science, Sungkyunkwan University, Suwon 440-746, Korea; marujunskku@gmail.com

**Keywords:** touch sensor, printable, conductive flexible adhesives, robotic application

## Abstract

Touch sensors are crucial in controlling robotic manipulation when a robot interacts with environmental objects. In this study, multilayer flexible touch sensors in the form of an array were developed. The sensors use ink-type conductive flexible adhesives as electrodes which were printed on polyethylene terephthalate (PET) films in a parallel equidistance stripe pattern. Between the two printed layers, a double-sided adhesive film was used to combine each layer and was perforated at the junctions of the top and bottom electrodes with different-sized circles. These holes represent switching mechanisms between the top and bottom electrodes, and their sizes make the sensor respond to different levels of external pressure. We showed the durability of the fabricated sensor with 1 mm diameter holes by repeated experiments of exerting normal pressure ranging from 0 to 159.15 kPa for 1000 cycles. In case of 1 mm diameter holes, the state of each sensor node was reliably determined by the threshold pressures of 127.3 kPa for increasing pressure and 111.4 kPa for decreasing pressure. On the other hand, decreasing the hole size from 3 to 0.5 mm caused an increase in the threshold pressure from 1.41 to 214 kPa. The relation between the hole size and the threshold pressure was analyzed by a mechanical model. The sensor performance was also verified on curved surfaces up to 60 mm radius of curvatures. Additionally, we fabricated a sensor with three levels of sensitivity with a conventional method which was a thermal evaporation to show the extendibility of the idea.

## 1. Introduction

Force feedback is important to control robotic manipulation, especially when a robotic manipulator interacts with unstructured environments or unknown objects [1]. In case of grasp motion of humanoids and surgical robots, it is necessary to measure not only contact force, but also contact location of the exerted force for grasping delicately with an end-effector [2,3,4]. Fingertip force torque sensors were used for measuring three dimensional force and torque to control the fingers that interacted with environment [5]. In other cases, a manipulator like a robot arm measured external force by using a sensitive skin in order to avoid collisions [6]. Using tactile sensors is one way to get the force information. There are four popular transducing strategies for the tactile sensors: capacitive, resistive, peizoresistive and optical [1].

Capacitive sensors consist of a pair of conductive plates and dielectric substance that separates the conductive plates. The external force causes a change in the gap between the conductive plates, which induce the change in capacitance [1]. Mannsfeld and Schwartz in Bao’s group reported highly sensitive flexible electronic skins formed by micro-structured capacitive sensors [7,8]. The sensor has high sensitivity due to the special micro-structured film. In general, the output signal of capacitive type sensors is small and susceptible to external noise such as parasitic capacitance. The disturbance caused by the parasitic capacitance is uncertain and tends to return wrong contact force sensing data. A special circuit is required to amplify the signal near the transducer and eliminate the effect of the parasitic capacitance between the sensor and objects.

Resistive sensors perceive mechanical deformation by measuring a change in resistance. Choi et al. reported a micro-machined flexible tactile sensor for three-axial loads detection [9]. A patterned sensing element such as a strain gauge was deposited on a polyimide film. The sensing element was covered with a single silicone bump to concentrate the force. Kim et al. introduced a highly sensitive resistive type single-axis tactile sensor with liquid pocket [10]. All layers in the sensor were made of silicone which is easy to deform by the external force because of its low elastic modulus. The liquid pocket was used to distribute the concentrated external force over the flexible sensing structure. Recently, liquid metal alloy has been used for wires in MEMS applications. Deformation of liquid metal shape induce the change in resistance. Park et al. fabricated soft artificial skin by using eutectic gallium-indium (eGaIn) [11]. Wong et al. used galinstan to fabricate flexible microfluidic normal force sensor skins [12].

The piezoresistive effect is a change in the electrical resistance caused by mechanical deformation of a material [1]. Several types of piezoresistive materials have been used for developing piezoresistive sensors (e.g., piezoresistive foam [13], piezoresistive rubber [14], etc.). Piezoresistive sensors have advantages of simple manufacturing processes, low noise, and low cost. Also some of them are commercially available. However, non-linear response and large hysteresis are disadvantages of piezoresistive sensors. Both resistive sensors and piezoresistive sensors also need a signal conditioning circuit such as an analog to digital converter [15], though their circuitry is much simpler than that of capacitive sensors. 

Optical sensors detect mechanical deformation based on the optical reflection between media with different refractive indices [1]. Kampmann et al. reported fiber-optic sensor arrays. The sensor consisted of light sources, optical fibers and camera chips [16]. Ohka et al. introduced an optical three-axis tactile sensor capable measuring normal and shearing force [17]. Optical sensors are highly sensitive, have high spatial resolution and are immune to electromagnetic interference, however, they are in generally bulky in size. Broader reviews of tactile sensing technologies are available in [1,18].

Signal conditioning circuitry is one of the issues for developing the tactile sensors. Sensor devices must provide signals that can be detected by readout electronics. Furthermore, scalability and flexibility are important features for a skin-like force sensor [14]. Especially, the flexibility is essential for operating the tactile sensors on any type of surface. However, it is difficult to extend the transducers into flexible sensor devices because embedding the flexible circuit into the sensor devices is not easy. In addition, initial state of those all flexible tactile sensors must be calibrated when curvature of the attaching surface changes. Because the sensors are affected by the initial geometric shape.

In this paper we present a flexible touch sensor which is fabricated with little effort by a printing method with ink-type conductive adhesives. A sensing cell in the sensor consists of a circular hole and two electrode layers, which constitutes a switching mechanism. The different sizes of the circular holes make the sensor respond to different levels of external pressure. The proposed sensor does not require any complex amplifying circuits due to this sensing mechanism. The complexity of the fabrication process is considerably reduced through the use of a printing method for electrode fabrication. This characterization is enhanced in mass production with scalability. Also, the touch sensor is sustainable on the various curved surfaces due to its flexibility. There is a limitation that the touch sensor can only provide low number of discretized force value measurements.

The remaining part of the paper is organized as follows: we show the design and the details of the fabrication process of the proposed sensor in Section 2, describe the experimental system and results in Section 3, and provide the conclusions in Section 4.

## 2. Experimental Section

### 2.1. Operation Principle

The working principle of the proposed sensor is simple. The proposed sensor is an array of sensing elements that can sense discrete pressures in accordance with their hole size. Each sensing element detects an on/off signal by an interconnection between top and bottom electrodes. These electrodes can be connected to each other through the holes in an intermediate layer. Applying external load on the sensing element causes connection between the top and bottom electrode, which makes resistance between the top and the bottom electrode decrease from the infinite to tens of ohm. Therefore, readout electronics can detect contact locations and pressures by reading the signal from I/O port. Sagisaka et al. proposed in [19] a large number of tactile sensing points in their conformable tactile sensing glove. Principle of the sensing mechanism is similar to ours in terms of that two electrode layers are connected by the external force. However, configuration of the sensor is totally different from that of our sensor.

### 2.2. Material

We previously reported highly conductive flexible adhesives (CFAs) that composed of silver (Ag) flakes, multiwalled carbon nanotubes decorated with silver nanoparticles (nAg-MWNTs), and nitrile butadiene rubber (NBR) [20,21]. NBR was used as a printable, flexible, and adhesive matrix and Ag flakes were used as primary conductive fillers. Carbon nanotubes with a high aspect ratio construct an effective electrical network between Ag flakes, and pre-attached silver nanoparticles improve the contact [20,21]. The conductivity is as high as 37,521 S/cm after the hot-rolling process that exhibited negligible variation upon bending [20]. In this study, CFA that was synthesized using a previously published protocol [20,21] was printed on a polyethylene terephthalate (PET) film substrate as an electrode.

### 2.3. Concept Design

**Figure 1:**
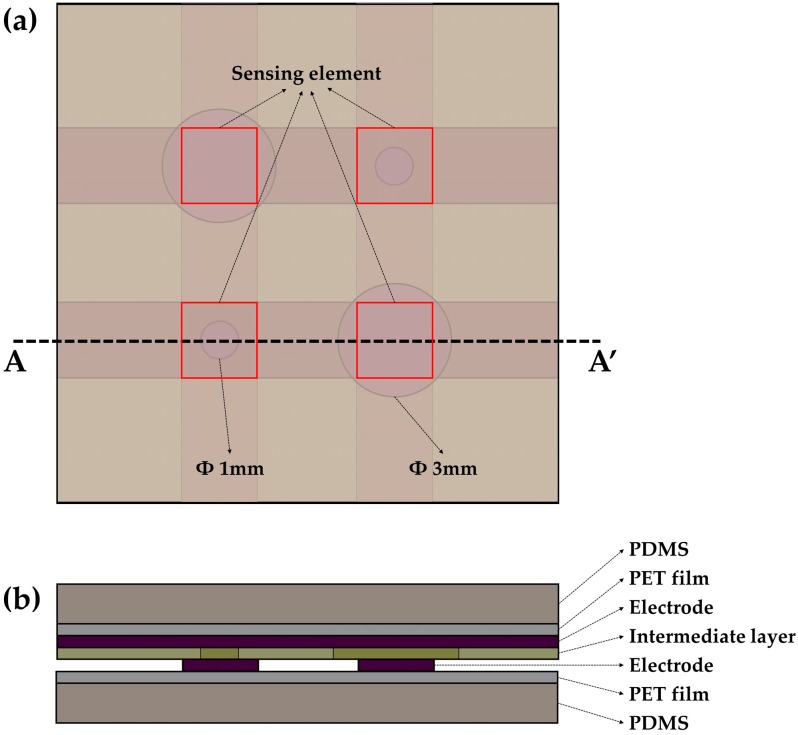
The structure of the sensor; (**a**) Top view; (**b**) Cross-sectional view along the AA’ line.

The proposed sensor consisted of three components which were an intermediate layer, a pair of electrode layers and a pair of cover layers with PDMS. The top and bottom electrode layers were arranged as shown in Figure 1a so that the top electrodes were horizontally and the bottom electrodes were vertically aligned in top view. Holes in the intermediate layer have two different sizes. The sizes of holes are 1 mm and 3 mm. Different levels of pressures were detected in accordance with the sizes of the holes. Figure 1b shows a cross-sectional view of the sensor along the AA’ line. The top and bottom electrodes were printed by CFAs (thickness 30 μm) and used as signal lines on the PET films (thickness 30 μm). The intermediate layer that was made by a double-sided adhesive film (adhesive 9 μm + PET 12 μm + adhesive 9 μm) was used as a bonding layer. In addition, the holes in the intermediate layer play a role in serving a gap between the top and the bottom electrodes. The cover layers with PDMS have 100 μm thickness. The electrode layers were encapsulated by the PDMS layers. These cover layers ensure durability and repeatability of the sensor due to protection of the primary sensing layers. 

### 2.4. Fabrication

**Figure 2:**
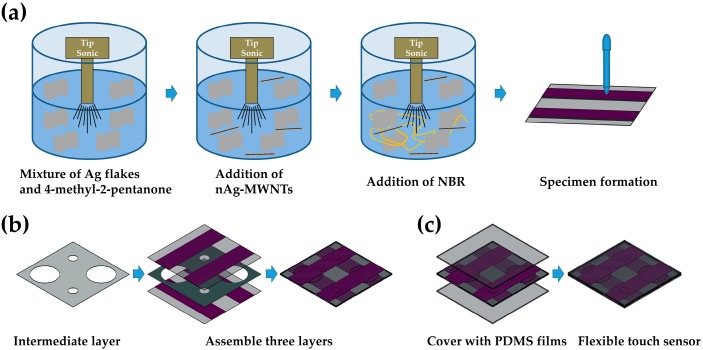
(**a**) Patterning the electrode with CFAs; (**b**) Assemble electrode layers; (**c**) Sealing with PDMS.

The proposed sensor is designed to be manufactured easily. Figure 2a shows the process of printing stripe pattern electrodes on a PET film. CFA was synthesized using a previously published protocol [20,21]. First, Ag flakes were added to 4-methyl-2-pentanone and stirred under ultrasonication. Next, nAg-MWNTs were added to the mixture and stirred. Lastly, NBR was added and stirred for one hour [20,21]. Then, the viscous ink (328 Pa·s) was printed on the PET films with a parallel stripe pattern. 

**Figure 3:**
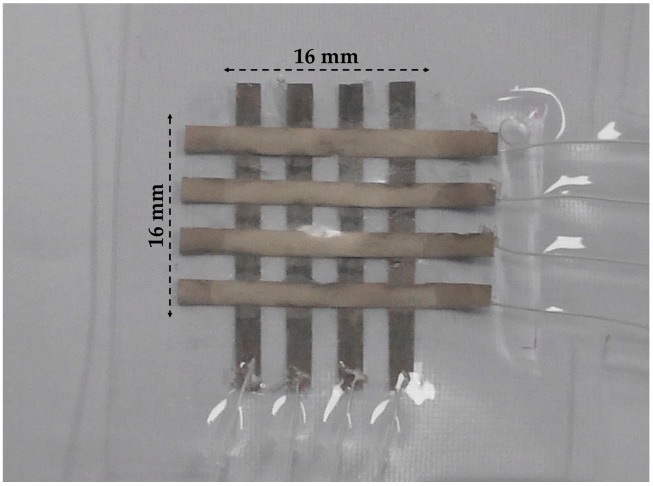
4 × 4 digital type tactile sensor.

The PET films used as the substrate for patterning the CFA had a thickness of 30 μm. A mask for the stripe pattern was formed on the PET film by attaching tapes with a pitch of 2 mm, where the clear adhesive tape has 30 μm in thickness and 2 mm in width. The tapes were lifted off after curing CFA resulting in a parallel stripe pattern. The intermediate layer was perforated by using hole punches. Diameter of the fabricated holes are 1 mm and 3 mm. The size of hole depends on the diameter of the hole punch. Various hole punches were used for creating different size of the holes. As shown in Figure 2b, the electrode layers were carefully assembled with the intermediate layer in order to align their electrodes perpendicularly like a cross. Wires for signal processing were attached on the electrodes with silver paste. PDMS (Sylgard 184, mix ratio 10:1 in weight, Dow Corning, Midland, MI, USA) solution was degassed in a centrifugal machine for 5 min. The PDMS was fabricated by a film applicator with a thickness of 100 μm. The PDMS films were cured in the oven at 140 °C for 15 min. These sensing layers were covered with the PDMS films for protection as shown in Figure 2c. Finally, the flexible touch sensor was assembled as shown in Figure 3. The sensor has a 4 × 4 array of sensing cells and its size is 16 mm × 16 mm. The size of the unit cell is 4 mm × 4 mm and spatial resolution of the sensor is 4 mm.

## 3. Results and Discussion

**Figure 4:**
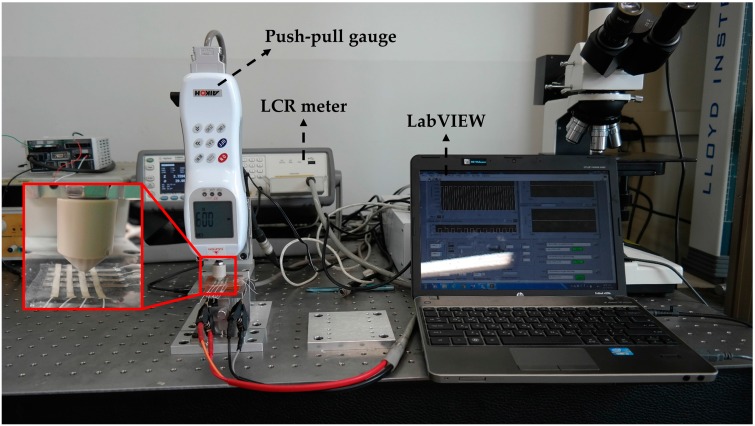
Experimental system.

An experimental system consisted of a push-pull gauge (RX-2, Aikoh, Osaka, Japan), a LCR meter (E4980A Precision LCR meter, Agilent, Santa Clara, CA, USA) and a laptop equipped with LabVIEW software (National Instruments, Austin, TX, USA) as shown in Figure 4. External load was applied on the sensor in a downward direction by the push-pull gauge. The push-pull gauge was mounted on the motorized linear guide. The force resolution of the push-pull gauge is 10 mN. Resistance of the sensor was measured by LCR meter with 1 V @ 100 kHz and Cp-Rp function. The measured resistance data was transmitted to the computer via a LAN cable. The LabVIEW software was used for collecting and saving the data. This software also, displayed the output data in real time. 

**Figure 5:**
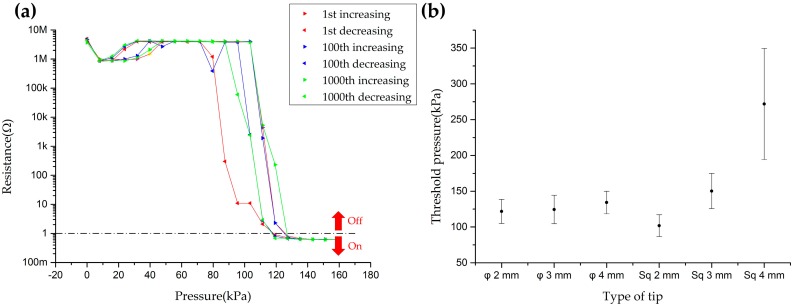
Performance of the sensor with a 1 mm diameter hole; (**a**) Resistance of a sensor versus pressure during 1000 exerting/releasing cycles with a circular-shaped flat Ø 4 mm tip; (**b**) Threshold pressures which are measured by various sizes of circular-shaped and square-shaped tips.

The performance of the cells with diameter 1 mm was verified as shown in Figure 5. First, the 1 mm diameter cell was tested by repeated experiments to check the durability and the hysteresis of the sensor. Normal pressure ranging from 0 to 160 kPa was exerted and released for 1000 cycles on the cell with a circular-shaped flat Ø 4 mm tip. The exerting/releasing cycle was automatically repeated by a motorized linear stage. In case of the 1st, 100th and 1000th exerting/releasing cycles, resistance values were recorded at specific pressures with a logarithmic scale as shown in Figure 5a. This result shows the sensor can endure a repeated mechanical stimulus more than 1000 times with low hysteresis. Criteria of on/off state was chosen as 1 Ω in order to describe threshold pressures. The threshold pressures of the 1 mm diameter cell were determined as 127.32 kPa with increasing force and 111.40 kPa with decreasing force. Next, effect of contact area on a single cell was tested. Four cells of the 1 mm diameter cells were performed by various tips: 2 mm × 2 mm, 3 mm × 3 mm and 4 mm × 4 mm square-shaped flat tips and Ø 2 mm, Ø 3 mm and Ø 4 mm circular-shaped flat tips. The sizes of the circular and square tips were chosen under 4 mm because the proposed sensor has 4 mm spatial resolution. Figure 5b shows the results of this experiment with average and standard deviation of threshold pressures. Except for the result of test that conducted with 4 mm × 4 mm square-shaped flat tip, average threshold pressures were in the range from 100 kPa to 150 kPa. The low spatial resolution can cause the threshold pressure to be off the trend like the experimental result of the threshold pressure with the 4 mm × 4 mm square-shaped flat tip. Furthermore, to figure out the maximum pressure tolerance of the proposed sensor, up to 20 N load (that is the maximum tolerance force of our push-pull gage) was exerted with the Ø 2 mm circular-shaped flat tip. The result was that the sensor endures the pressure over 6.36 MPa, which exceeds our interested pressure range, approximately 35–220 kPa (15–90 g·wt. [18]). 

**Figure 6:**
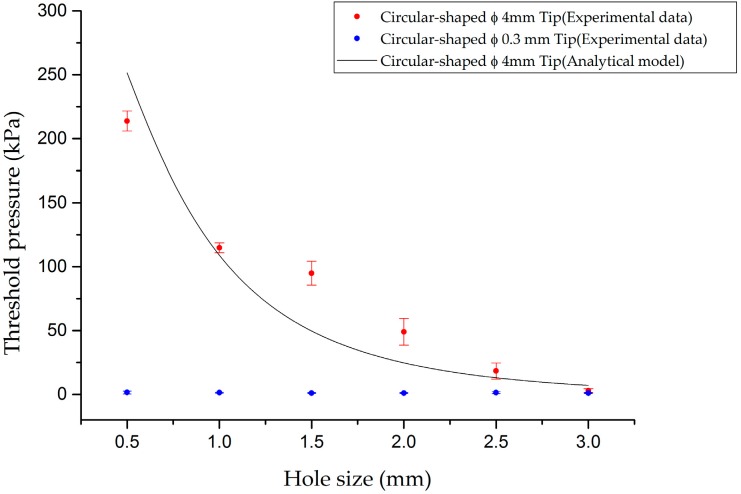
Relation between the threshold pressure and the hole size. The experimental results with the circular-shaped Ø 0.3 mm tip and Ø 4 mm tip are shown. The simple analytical model that is fitted with the circular-shaped Ø 4 mm tip test result is shown.

To demonstrate the relation between the hole size and the threshold pressure, a sensor that consists of six cells with different hole sizes was tested; the hole diameters were from 0.5 mm to 3.0 mm with 0.5 mm intervals. Three trials were conducted with each cell to obtain threshold pressures. For this experiment we used two circular-shaped flat tips with Ø 0.3 mm and Ø 4 mm diameters. As shown in Figure 6, when the load was applied with the circular-shaped flat Ø 0.3 mm tip whose diameter is smaller than the minimum hole size, the threshold pressures of all cells were around 2 kPa. On the other hand, when the cell was exerted with the circular Ø 4 mm tip whose diameter is larger than the maximum hole size, threshold pressure of each cell increased from 2.91 kPa to 214 kPa as size of the holes decreased.

**Figure 7:**
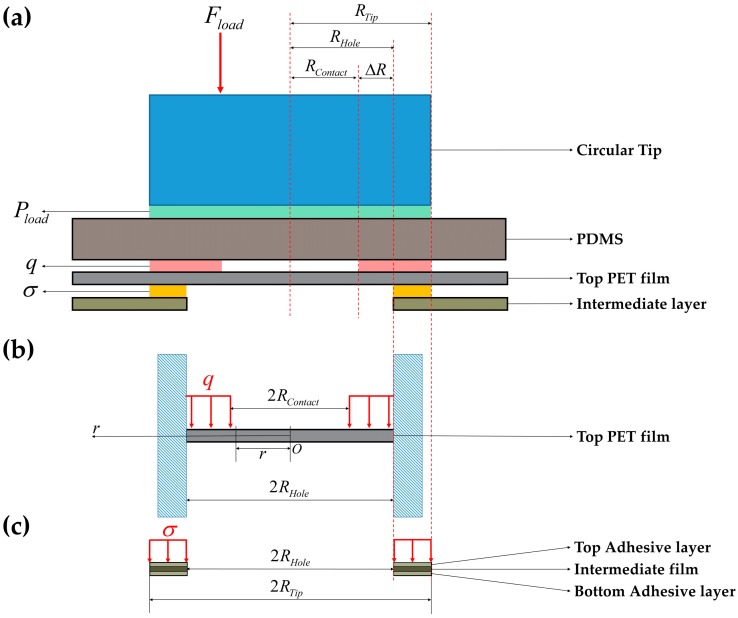
Force diagram for the analytical model of the printable flexible touch sensor. *P_Load_* represents the uniformly distributed pressure on PDMS, *q* represents the uniformly distributed pressure on upper PET film and *σ* represents the uniformly distributed pressure on intermediate layer; (**a**) Simplified model for the upper half of the sensor; (**b**) Simplified model for deflection of the top PET film; (**c**) Simplified model for deformation of the top adhesive layer, where the double-sided adhesive film consists of the top adhesive layer, the intermediate film and the bottom adhesive layer.

We present a simple analytical model for the relation between the hole size and threshold pressure by simplifying and some assumptions. We assume that the diameter of the circular tip is larger than the hole size. A Young’s modulus of PET film is about 2.3 GPa [22] and Young’s modulus of PDMS was measured as 1.189 MPa. The PET film and PDMS film are detachable, because they were not bonded together in fabrication. Because the PET film is even stiffer than the PDMS film and they are detachable, it is obvious that the pressure is distributed on the deformable region of the PET film as shown in Figure 7a. To simplify the model, we did not consider electrodes and assumed that the exerted pressure is uniformly distributed over the PET film as follows:

(1) $$q=\frac{P_{Load}R_{Tip}^{2}}{\left(R_{Tip}^{2}-R_{Contact}^{2}\right)}$$

where $R_{Tip}$ is the radius of the circular-shaped flat Ø 4 mm tip and $R_{Contact}$ is the radius of the circular boundary between the loaded and the unloaded region in the PET film. 

According to the theory of the plates, deflection of axis-symmetry circular plates is obtained from the following:

(2) $$\frac{d}{dr}\left[\frac{1}{r}\frac{d}{dr}\left(r\frac{dw_{PET}}{dr}\right)\right]=\frac{Q}{D}$$

(3) $$M_{r}=-D\left(\frac{d^{2}w_{PET}}{dr^{2}}+\frac{\nu_{PET}}{r}\frac{dw_{PET}}{dr}\right)$$

(4) $$D=\frac{1}{12}\frac{E_{PET}h^{3}}{\left(1-{\nu_{PET}}^{2}\right)}$$

where, r is radial distances of points in the middle plane of PET film, $w_{PET}$ is the deflections in the downward direction, $M_{r}$ is bending moment per unit length, Q is shearing force per unit length and D is flexural rigidity [23]. In Equation (4), the PET film properties are as follows: thickness h=30 μm, Young’s modulus $E_{PET}=2.3\text{ GPa}$ [22] and Poisson’s ratio $\nu_{PET}=\;0.3$ [24]. By the force analysis in Figure 7b, the shearing force is obtained as shown in Equation (5):

(5) $$\{\begin{array}{ll} Q=0 & \left(0\leq r\leq R_{Contact}\right)\\ Q=\frac{q\left(r^{2}-R_{Contact}^{2}\right)}{2r} & \left(R_{Contact}\leq r\leq R_{Hole}\right) \end{array}$$

Using Equations (2) and (5), w, $\frac{dw}{dr}$ and $\frac{d^{2}w}{dr^{2}}$ were calculated as follows:

(6) $$\{\begin{array}{ll} w_{PET}=-\frac{c_{1}r^{2}}{4}-c_{2}\ln r+c_{3} & \left(0\leq r\leq R_{Contact}\right)\\ w_{PET}=\frac{qr^{4}}{64D}-\frac{qR_{Contact}^{2}}{8D}r^{2}\ln\frac{r}{R_{Hole}}+\frac{qR_{Contact}^{2}r^{2}}{8D}-\frac{c_{4}}{4}r^{2}-c_{5}\ln\frac{r}{R_{Hole}}+c_{6} & \left(R_{Contact}\leq r\leq R_{Hole}\right) \end{array}$$

(7) $$\{\begin{array}{ll} \frac{dw_{PET}}{dr}=-\frac{c_{1}r}{2}-c_{2}\frac{1}{r} & \left(0\leq r\leq R_{Contact}\right)\\ \frac{dw_{PET}}{dr}=\frac{qr^{3}}{16D}-\frac{qR_{Contact}^{2}}{4D}r\ln\frac{r}{R_{Hole}}+\frac{qR_{Contact}^{2}}{8D}r-\frac{c_{4}}{2}r-\frac{c_{5}}{r} & \left(R_{Contact}\leq r\leq R_{Hole}\right) \end{array}$$

(8) $$\{\begin{array}{ll} \frac{d^{2}w_{PET}}{dr^{2}}=-\frac{c_{1}}{2}+c_{2}\frac{1}{r^{2}} & \left(0\leq r\leq R_{Contact}\right)\\ \frac{d^{2}w_{PET}}{dr^{2}}=\frac{3qr^{3}}{16D}-\frac{qR_{Contact}^{2}}{4D}\ln\frac{r}{R_{Hole}}-\frac{qR_{Contact}^{2}}{8D}-\frac{c_{4}}{2}+\frac{c_{5}}{r^{2}} & \left(R_{Contact}\leq r\leq R_{Hole}\right) \end{array}$$

where $c_{i}\;(i=1,2,\cdots ,6)$ are integral coefficients. The boundary conditions for edge of the PET film are clamped edge and can be represented as Equation (9):

(9) $$\begin{array}{l} {w_{PET}|}_{r=R_{Hole}}=0\\ {\frac{dw_{PET}}{dr}|}_{r=R_{Hole}}=0 \end{array}$$

The deflection should be continuous and smooth, so the values of $\frac{dw_{PET}}{dr}$ and $w_{PET}$ are identical to zero at $r=R_{Contact}$, and additionally $\frac{dw_{PET}}{dr}=0$ at r=0. Also the bending moment $M_{r}$ should be equal at $r=R_{Contact}$. Using these conditions and Equations (3) and (6)–(9), integral coefficients were determined as follows:

(10) $$\begin{array}{l} c_{1}=\frac{qR_{Hole}^{2}}{8D}+\frac{qR_{Contact}^{2}}{2D}\ln\frac{R_{Contact}}{R_{Hole}}-\frac{qR_{Contact}^{4}}{8DR_{Contact}^{2}}\\ c_{2}=0\\ c_{3}=\frac{qR_{Hole}^{4}}{64D}-\frac{qR_{Hole}^{2}R_{Contact}^{2}}{16D}+\frac{3qR_{Contact}^{4}}{64D}-\frac{qR_{Contact}^{4}}{16D}\ln\frac{R_{Contact}}{R_{Hole}}\\ c_{4}=\frac{qR_{Hole}^{2}}{8D}+\frac{qR_{Contact}^{2}}{4D}-\frac{qR_{Contact}^{4}}{8DR_{Hole}^{2}}\\ c_{5}=\frac{qR_{Contact}^{4}}{16D}\\ c_{6}=\frac{qR_{Hole}^{4}}{64D}-\frac{qR_{Hole}^{2}R_{Contact}^{2}}{16D}-\frac{qR_{Contact}^{4}}{32D} \end{array}$$

Thus, the maximum deflection occurs at r=0 and it was obtained as Equation (11):

(11) $$w_{PET,\max}=\frac{P_{Load}R_{Tip}^{2}}{64D\left(R_{Tip}^{2}-R_{Contact}^{2}\right)}\left(R_{Hole}^{4}-4R_{Hole}^{2}R_{Contact}^{2}+3R_{Contact}^{4}-4R_{Contact}^{4}\ln\left(\frac{R_{Contact}}{R_{Hole}}\right)\right)$$

Equation (11) is the result when adhesive layers in Figure 7c are treated as a rigid body. However, deformation of the adhesive layers causes additional deflection of the PET film. This deformation was also simplified as shown in Figure 7c. In this model, uniformly distributed pressure which is applied on the adhesive layer is as follows:

(12) $$\sigma =\frac{P_{Load}R_{Tip}^{2}}{\left(R_{Tip}^{2}-R_{Hole}^{2}\right)}$$

The deformation of the adhesive layer was calculated by well-known Hooke’s law:

(13) σ=Eε

(14) $$\varepsilon =\frac{\Delta t_{Adhesive}}{t_{Adhesive}}$$

where $t_{Adhesive}$ is the thickness of the adhesive layers and $\Delta t_{Adhesive}$ is the change in the thickness. $t_{Adhesive}$ is 9 μm when the sensor is not loaded. We assumed that the additional deflection $w_{Adhesive}$ and the deformation of the adhesive layer $\Delta t_{Adhesive}$ are roughly similar to:

(15) $$w_{Adhesive}\approx \Delta t_{Adhesive}$$

Using Equations (11)–(15), the additional deflection was obtained as follows:

(16) $$w_{Adhesive}\approx \frac{P_{Load}R_{Tip}^{2}t_{Adhesive}}{\left(R_{Tip}^{2}-R_{Hole}^{2}\right)E_{Adhesive}}$$

The proposed sensor is symmetric with respect to the intermediate layer, which means that the deformation of the lower layers and upper layers are the same. When a total deformation $w_{Total}$, the sum of these deformation, is equal to 30 μm, two electrodes are connected, therefore we have that:

(17) $$w_{Total}=2w_{PET}+2w_{Adhesive}$$

According to Equations (11), (16) and (17), the threshold pressure was obtained as follows:

(18) $$P_{Load}=\frac{w_{Total}}{R_{Tip}^{2}\left(\frac{2t_{Adhesive}}{E_{Adhesive}\left(R_{Tip}^{2}-R_{Hole}^{2}\right)}+\frac{R_{Hole}^{4}-4R_{Hole}^{2}R_{Contact}^{2}+3R_{Contact}^{4}-4R_{Contact}^{4}\ln\left(\frac{R_{Contact}}{R_{Hole}}\right)}{32D\left(R_{Tip}^{2}-R_{Contact}^{2}\right)}\right)}$$

ΔR (mm) represents the contact region between PET film and PDMS film, and is equal to $R_{Hole}-R_{Contact}$ as shown in Figure 7a. We assume that Δ*R* (mm) increases linearly from 0.25 mm to *a* (mm) as *R_Hole_* increases from 0.25 mm to 3 mm as follows:

(19) $$\Delta R=\frac{a-0.25}{1.5-0.25}(R_{Hole}-0.25)+0.25\;(0.25\leq \Delta R\leq a)$$

Young’s modulus of the adhesive in Equation (18) and constant *a* in Equation (19) are unknown. Therefore the simple analytical model was fitted with these two constant variables by using the least-squares percentage regression [25]. The least-squares percentage regression is a method that minimizes the sum of relative errors, which is:

(20) $$S=\sum {\left(\frac{P_{Data}-P_{Model}}{P_{Data}}\right)}^{2}$$

As the adhesive layer is predicted to be softer than the PDMS, $E_{Adhesive}$ was chosen in the range of 100–800 kPa with the interval of 1 kPa. The constant *a* was chosen in the range of 0.25–0.4 mm with the interval of $1.5\times 10^{-4}$ mm. As $R_{Contact}^{4}\ln\left(\frac{R_{Contact}}{R_{Hole}}\right)$ does not exist at Δ*R* = 0.25 mm, all terms of 0.25 mm in Equation (19) were changed to 0.24999 for computation. By complete enumeration survey in the previous range, the constants obtained: *a* was 0.3174 and *E_Adhesive_* was 186 kPa with 0.9406 of *S*. This result is shown in Figure 6. Thus, the threshold pressure is a function of the hole size. The threshold pressure deceases as the size of the hole continues to increase.

**Figure 8:**
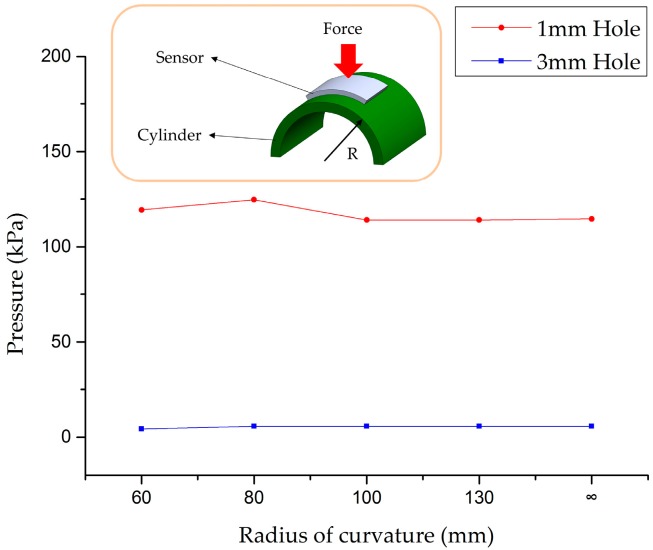
Threshold pressure according to the radius of curvature.

The proposed tactile sensor was tested on curved surfaces. The threshold pressure of sensors with 1 mm diameter and 3 mm diameter holes was measured on the various curved surfaces as shown in Figure 8. The tactile sensor was placed on the surface of a cylindrical rod. Then, normal pressure was exerted on the single cell by the circular-shaped Ø 4 mm flat tip. The threshold pressure was almost invariant in each cell as the radius of curvature decreases up to 60 mm. This result is shown in Figure 8. 

**Figure 9:**
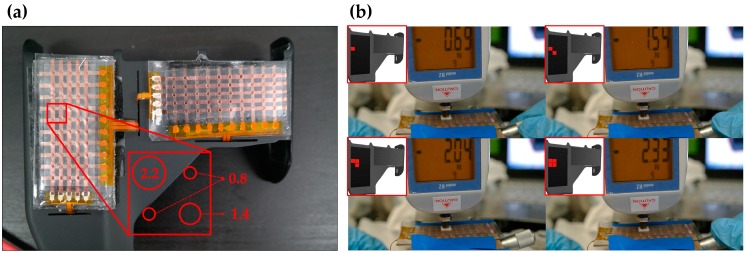
The digital type tactile sensor; (**a**) A photo of the extended sensor; (**b**) The various force responses according to the hole size.

As discussed, the threshold pressure decreased with increasing hole size. This mechanism can be easily extended to multiple force level touch sensors which we call digital type touch sensors as shown in Figure 9a. Electrodes of this digital type touch sensor were fabricated with Cu/Cr by the thermal evaporation method to show that the design concept can be applied with conventional fabrication methods. To increase sensitivity and reliability, we implemented cylindrical bumper structure with 1.5 mm diameter and 0.2 mm height in the top PDMS layer. The cylindrical bumper structure was vertically aligned between the top PDMS layer and the bottom PET film on each cell. The intermediate layer of the extended sensor has three different hole diameters, 2.2 mm, 1.4 mm and 0.8 mm. The force was applied on four cells at the same time using a square-shaped flat 20 mm × 20 mm tip. In accordance with the sizes of the holes, the threshold force in each cell was 0.69 N, 1.54 N, and 2.04–2.33 N as shown in Figure 9b.

## 4. Conclusions/Outlook

In this paper, a 4 × 4 array flexible touch sensor has been designed and fabricated by a printing method. The sensor was composed of an intermediate layer, a pair of electrode layers and a pair of PDMS layers. A circular hole in the intermediate layer and two electrodes in each electrode layer compose a sensing cell. Connecting the two electrodes, a loaded pressure can be detected by measuring the dramatic change in resistance. The threshold pressure deceases as the size of the hole increases. Using this characteristic, the sensor can be fabricated to measure multi-step pressures by altering the hole-size, although each sensing cell of the sensor is designed to detect only one step pressure output. This mechanism has an advantage in that it does not require additional complex circuits. The electrodes of the sensor were made of CFAs which are based on the rubber. CFAs were printed on PET film with a typical stencil method. CFAs have good performance regarding flexibility, conductivity and durability. The proposed sensor has a good flexibility that enables the sensor to perform on various curved surfaces. As the proposed idea is easily scalable to measure multiple discrete levels of forces with accurate measurements of contact locations, it has potential to be used in robotic applications. 

## References

[1] Kappassov Z., Corrales J.-A., Perdereau V. (2015). Tactile sensing in dexterous robot hands—Review. Robot. Auton. Syst..

[2] Tiwana M.I., Redmond S.J., Lovell N.H. (2012). A review of tactile sensing technologies with applications in biomedical engineering. Sens. Actuators A Phys..

[3] Kawasaki H., Komatsu T., Uchiyama K. (2002). Dexterous anthropomorphic robot hand with distributed tactile sensor: Gifu hand II. IEEE ASME Trans. Mech..

[4] Wong R.D.P., Hellman R.B., Santos V.J. (2014). Haptic Exploration of Fingertip-Sized Geometric Features Using a Multimodal Tactile Sensor. Proc. SPIE.

[5] Butterfass J., Grebenstein M., Liu H., Hirzinger G. DLR-Hand II: Next Generation of a Dextrous Robot Hand. Proceedings of the IEEE International Conference on Robotics and Automation (2001 ICRA).

[6] Lumelsky V.J., Cheung E. (1993). Real-time collision avoidance in teleoperated whole-sensitive robot arm manipulators. IEEE Trans. Syst. Man Cybern..

[7] Mannsfeld S.C., Tee B.C., Stoltenberg R.M., Chen C.V.H., Barman S., Muir B.V., Sokolov A.N., Reese C., Bao Z. (2010). Highly sensitive flexible pressure sensors with microstructured rubber dielectric layers. Nat. Mater..

[8] Schwartz G., Tee B.C.-K., Mei J., Appleton A.L., Kim D.H., Wang H., Bao Z. (2013). Flexible polymer transistors with high pressure sensitivity for application in electronic skin and health monitoring. Nat. Commun..

[9] Choi W.-C. (2010). Polymer micromachined flexible tactile sensor for three-axial loads detection. Trans. Electr. Electron. Mater..

[10] Kim S., Kim B.C., Jung J., Koo J.C., Choi H.R., Moon H. (2014). Highly Sensitive Resistive Type Single-Axis Tactile Sensor with Liquid Pocket. Proc. SPIE.

[11] Park Y.L., Chen B.R., Wood R.J. (2012). Design and fabrication of soft artificial skin using embedded microchannels and liquid conductors. IEEE Sens. J..

[12] Ponce Wong R.D., Posner J.D., Santos V.J. (2012). Flexible microfluidic normal force sensor skin for tactile feedback. Sens. Actuators A Phys..

[13] Weiss K., Worn H. The Working Principle of Resistive Tactile Sensor Cells. Proceedings of the IEEE International Conference on Mechatronics and Automation.

[14] Drimus A., Kootstra G., Bilberg A., Kragic D. (2014). Design of a flexible tactile sensor for classification of rigid and deformable objects. Robot. Auton. Syst..

[15] Oddo C.M., Beccai L., Muscolo G.G., Carrozza M.C. A Biomimetic Mems-Based Tactile Sensor Array with Fingerprints Integrated in a Robotic Fingertip for Artificial Roughness Encoding. Proceedings of the IEEE International Conference on Robotics and Biomimetics.

[16] Kampmann P., Kirchner F. (2014). Integration of fiber-optic sensor arrays into a multi-modal tactile sensor processing system for robotic end-effectors. Sensors.

[17] Ohka M., Kobayashi H., Takata J., Mitsuya Y. Sensing Precision of an Optical Three-Axis Tactile Sensor for a Robotic Finger. Proceeding of the 15th IEEE International Symposium on Robot and Human Interactive Communication.

[18] Dahiya R.S., Metta G., Valle M., Sandini G. (2010). Tactile sensing—From humans to humanoids. IEEE Trans. Robot..

[19] Sagisaka T., Ohmura Y., Kuniyoshi Y., Nagakubo A., Ozaki K. High-Density Conformable Tactile Sensing Glove. Proceedings of the 2011 11th IEEE-RAS International Conference on Humanoid Robots.

[20] Ma R., Kwon S., Zheng Q., Kwon H.Y., Kim J.I., Choi H.R., Baik S. (2012). Carbon-nanotube/silver networks in nitrile butadiene rubber for highly conductive flexible adhesives. Adv. Mater..

[21] Kwon S., Ma R., Kim U., Choi H.R., Baik S. (2014). Flexible electromagnetic interference shields made of silver flakes, carbon nanotubes and nitrile butadiene rubber. Carbon.

[22] Gupta S., Dixit M., Sharma K., Saxena N. (2009). Mechanical study of metallized polyethylene terephthalate (pet) films. Surf. Coat. Technol..

[23] Timoshenko S., Woinowsky-Krieger S. (1959). Theory of Plates and Shells.

[24] Ma T., Bhushan B., Murooka H., Kobayashi I., Osawa T. (2002). A novel technique to measure the poisson’s ratio and submicron lateral dimensional changes of ultrathin polymeric films. Rev. Sci. Instrum..

[25] Tofallis C. (2009). Least squares percentage regression. J. Mod. Appl. Stat. Methods.

